# Production, Control, and Visual Guidance of Saccadic Eye Movements

**DOI:** 10.1155/2013/752384

**Published:** 2013-10-23

**Authors:** Jeffrey D. Schall

**Affiliations:** Department of Psychology, Center for Integrative & Cognitive Neuroscience, Vanderbilt Vision Research Center, Vanderbilt University, Nashville, TN 37240, USA

## Abstract

Primate vision is served by rapid shifts of gaze called saccades. This review will survey current knowledge and particular problems concerning the neural control and guidance of gaze shifts.

## 1. Introduction

Being primates endowed with a fovea providing acute vision over a very small range of the visual field, we must shift gaze to explore the world. Rapid eye movements called saccades direct the line of sight onto objects of interest in the visual field, often conspicuous objects like a berry among leaves and sometimes important objects like the family member among a social group. More is understood about visually guided saccade production than any other sensory motor system for several reasons. First, movements of the eyes are simpler than movements of the limbs or vocal apparatus because they have fewer degrees of freedom and can ignore gravity. Second, every neuron from the sensory through the motor is accessible to inquiry within the cranium. Third, advances in technology have provided accurate measurements and manipulations of the fine details of eye movements. 

Eye movement research with macaque monkeys has profoundly influenced clinical neurology and ophthalmology, and this translational interface runs both directions. On the one hand, insights from monkey studies have been essential for clinicians to interpret neurological examinations. On the other hand, properties of human eye movements have stimulated neurophysiological studies that have, in turn, informed clinical practice. While the neural control of movements is certainly instantiated through molecular mechanisms, it has become clear that knowledge at the level of neural systems is most useful for this clinical translation. For example, monkey models of strabismus and amblyopia (e.g., [1–5]), fourth nerve palsy (e.g., [6]), nystagmus (e.g., [7, 8]), and Parkinson's disease (e.g., [9, 10]) have provided precise information that would otherwise have been left to clinical guesswork. These monkey models have furthermore provided refinements of new treatments such as deep brain stimulation for Parkinson's disease, optical treatments for developmental strabismus, and drugs for nystagmus. Similarly, many neuropsychiatric disorders are associated with problems of gaze control (e.g., [11]), so obtaining neurophysiological data from monkeys performing tasks in which these problems are expressed by patients (and their relatives) will provide information that can improve the diagnosis and possibly treatment of these disorders. 

The literature on the production, guidance, and effects of saccades is very broad. A PubMed search in July 2013 with the keyword “saccade” resulted in >9000 publications. Publications about saccades appeared at a relatively low rate (<50/year) until the 1990s whereupon the publication rate increased dramatically to a level of ~500/year. Such a vast literature cannot be surveyed here, but comprehensive reviews have appeared recently (e.g., [12–14]). This review will focus on new developments in our understanding of how the brain controls the initiation and guides the endpoint of saccadic eye movements. 

Space does not permit reviewing fascinating new research on the relationships between vision and saccades, so the interested reader is pointed to the body of research demonstrating that gaze tends to focus on conspicuous and informative features of an image during scrutiny of simple geometric stimuli (e.g., Liversedge and Findlay 2000), natural images (e.g., [15]) or text (e.g., [16]), and during complex natural behaviors (e.g., [17–19]) leading to the hypothesis that gaze can be directed in a statistically optimal manner (e.g., Najemnik and Geisler, 2009). The reader should also be alerted to the renewed interest and continuing disagreements about the effects, utility, and production of microsaccades (<1/5°) in relation to vision (e.g., [20]; Martinez-Conde & Macknik 2011) and attention (e.g., [21–23]). We also will not review the literature investigating how saccades influence vision beyond noting that as you can learn by watching yourself shift gaze in a mirror between left and right eyes, and we experience phenomenal blindness during saccades in part because of visual masking and in part because the responsiveness of neurons in the visual pathway is attenuated during saccades (e.g., [24, 25]). In laboratory testing, visual perception of location and spatial relations is systematically distorted immediately before, during, and immediately after saccades (e.g., [26]) presumably due to shifts of the visual field representation coinciding with saccade generation (e.g., [27, 28]). The stability of visual perception that we experience even though we are shifting gaze two or three times each second has been explained as the consequence of an efference copy signal [29] that recent physiological research has mapped through the visuomotor pathway [19]. We should note the more recent research has found that these effects are attenuated when multiple objects are presented (e.g., [30]), so the generality of the laboratory findings with single spots of light presented at predictable locations for vision in crowded natural environments requires further investigation.

## 2. Saccade Production

The biomechanical and neural processes in the brainstem producing saccades have been described in detail (reviewed by [31, 32]). Recent years have witnessed important insights into the complexity of the oculomotor periphery. These include the organization of the extraocular muscles into functionally distinct fiber groups and the presence of connective tissue pulleys that change the pulling directions of rectus muscles, so that the eye's rotational axis varies with eye position to accomplish Listing's law. It is now possible to characterize the motor neurons innervating different muscle fibers types (e.g., [33]) and measure innervation and forces simultaneously (e.g., [34]).

Saccadic eye movements are initiated when a pulse of force is produced through the high-frequency discharge of oculomotor neurons innervating the extraocular muscles. The pulse of force overcomes the viscoelastic forces acting against ocular rotation. Eye position is maintained at eccentric angles by a step of force produced through sustained discharge of oculomotor neurons. Saccadic eye movements are characterized by a very precise relationship between amplitude, velocity, and duration. This relationship is achieved through a circuit in the brainstem consisting of burst neurons that provide the burst of action potentials to the oculomotor neurons to produce ipsiversive saccades; the magnitude of the burst scales with eye velocity for saccades is less than ~20°. The circuit also includes tonic neurons that innervate the oculomotor neurons and are innervated by the burst neurons and are understood to perform velocity to position integration that provides the step of force needed to maintain eccentric gaze. The details of this integration process have occupied considerable attention in recent years (e.g., [35, 36]).

Burst neuron activation is gated by omnipause neurons (OPNs) and inhibitory burst neurons (IBNs), so that initiation of a saccade requires inhibition of the omnipause neurons (e.g., [37]). This inhibition has been described through intracellular recordings [38] and more recently through LFPs [39]. It begins as an abrupt hyperpolarization, controlled more by glycinergic than GABAergic inputs [40] that is sustained until the saccade is completed. The inhibition on omnipause neurons has multiple sources including long-lead burst neurons in the brainstem, the superior colliculus, the frontal eye field, and the supplementary eye field. IBNs receive monosynaptic excitation from contralateral SC sites producing saccades of all vectors and disynaptic inhibition from the ipsilateral SC via contralateral IBNs. OPNs receive excitation from the rostral end of contralateral and ipsilateral SC and disynaptic inhibition from the caudal SC mainly via IBNs [37]. 

While the neural processes responsible for initiating and producing saccades are reasonably well understood, the mechanism responsible for terminating saccades is less certain. Research on this problem has been guided by the engineering principles of feedback control systems [41]. The received view is that the burst neurons are driven by a dynamic motor error signal that is the difference between current and desired eye position (or displacement). Evidence for a feedback control mechanism seems beyond dispute. Experimental activation of OPN while saccades are in flight can result in arrested velocity, but when the stimulation is removed, the saccade continues to completion, fulfilling the motor error. 

How this comparison is accomplished in the feedback loop remains uncertain. Key questions center on whether the error signal is eye position, eye displacement in the current saccade or even gaze (eye + head), and also the anatomical substrate of the comparator. It seems unlikely that natural reactivation of OPN terminate saccades because the duration of the OPN pause does not correlate well with saccade duration; normal saccades can be produced after OPN lesions and patients with diseases that cause abnormal saccade durations exhibit high-frequency conjugate oscillations following saccades indicative of OPN inactivation (e.g., [42]). One hypothesis proposed that the SC is in the dynamic motor error feedback loop through a pattern of spatiotemporal dynamics of activation moving from the location representing the vector of the saccade to the rostral end of the SC that was supposed to engage active fixation (e.g., [43]). Evidence against this hypothesis (e.g., [44]) has shifted attention to the cerebellum that is necessary for adapting the amplitude and duration of saccades across conditions (e.g., [45]). 

## 3. Control of Saccade Initiation

We must shift gaze to see things in our environment, but vision is impaired during saccades, so the brain must balance these competing constraints. In this section, we will survey how the brain prepares and initiates saccades and how those processes may be adjusted by other brain systems that monitor the consequences of actions. 

### 3.1. Direct Control

Direct control will refer to the processes that specify the response time (RT). These processes can vary with task demands and context. For example, when given a warning (“ready”) before an imperative trigger signal (“go”), subjects respond earlier and more reliably than when no warning is given (Niemi & Näätänen 1981). Also, saccade RT is influenced by repetition of stimuli or responses and by the history of reinforcement (e.g., Dorris et al. 1999; [46, 47]). This variation can be explained in terms of a process that transpires after the warning signal that leads to faster responses and is influenced by events in preceding trials to influence the readiness to initiate a movement. We will refer to this process as *response preparation*. Further evidence for response preparation is the observation that partially prepared responses are more difficult to withhold if an imperative “stop” signal occurs later in time (e.g., Logan & Cowan 1984; Hanes & Schall 1995).

The connections between these preparatory processes and the events that trigger a saccade are not understood. We know that the OPNs are not modulated at all during periods of saccade preparation (Everling et al. 1998). Most models of the brainstem mechanisms of saccade generation do not address the question of what turns off the omnipause neurons to release inhibition on the burst neurons that will generate the pulse through the oculomotor neurons (reviewed by [48]). In some models, it just happens (e.g., [41, 49]), and in others it is related to the specification of a motor error signal (e.g., [50–52]). The original models were not concerned with explaining the variation of saccade initiation time, but in subsequent models, the events that ultimately inhibit the omnipause neurons are related to processes occurring in the superior colliculus, basal ganglia, thalamus, and cerebral cortex (e.g., [53–56]). The latest models were inspired by the observation that the dynamics of the activity of specific neurons in the FEF, and SC accounts for the variation of saccade initiation time. Saccades are initiated when the discharge rate of presaccadic movement neurons in FEF and SC reaches a particular threshold (e.g., [57]; Hanes & Schall 1996). The variation of saccade latency in a range of tasks calling for speeded responses is accounted for by the time taken to reach that threshold; the variation in time to threshold arises from randomness in the rate of growth (Hanes & Schall 1996; Ratcliff et al. 2003, 2007) [58, 59] although other studies in other task conditions find variability of the baseline activity as well (Dorris et al. 1997) [58] and also systematic changes in the onset of the accumulation when it takes longer to locate the target [60] or adjust to task conditions [61]. This variable accumulation to a threshold inspired the identification of this activity with the process described by stochastic accumulator models developed by cognitive psychologists (e.g., [62]).

The neural control of movement initiation has been investigated fruitfully using the stop signal (or countermanding) task. Developed to investigate human performance, the countermanding paradigm probes a subject's ability to control the initiation of movements by infrequently presenting an imperative stop signal in a response time task (reviewed by [63]). This task is diagnostic of disorders of impulse control and response monitoring (e.g., [64–66]). The subjects' task is to produce a saccade as quickly as possible after a target appears to cancel that partially prepared saccade if a stop signal is presented; the stop signal was the reappearance of the fixation spot (Hanes & Schall, 1995). Performance of this task can be understood as the outcome of a race between a process with random finish times that generates the movement (GO process) and another random process that cancels the movement (STOP process) (Logan and Cowan 1984). Under reasonable assumptions, the duration of the covert STOP process can be derived from the proportion of successful stop trials and the RT on trials with no stop signal (finish time of overt GO process). The duration of the STOP process is referred to as *stop signal reaction time*; it measures the time needed to cancel the planned movement. 

The validity of SSRT as a measure of the time to interrupt movement preparation and execution has been tested using various approaches. For example, saccades can be elicited prematurely by delivering an air puff to the eye that causes an eyelid blink that inhibits omnipause neurons. When monkeys performed the stop signal task, air puffs presented more than ~70 ms after the stop signal rarely evoked saccades, and the saccades triggered close to SSRT tended to be hypometric [67]. Also, during a combined eye-head gaze countermanding task, a burst of antagonist neck muscle activity was observed on stop signal trials when subjects initiated small head movements even though gaze remained stable due to the vestibular ocular reflex (Goonetilleke et al. 2010). This “braking” pulse only occurred when the head movement was interrupted in midflight and was concomitant with SSRT. 

**Figure 1:**
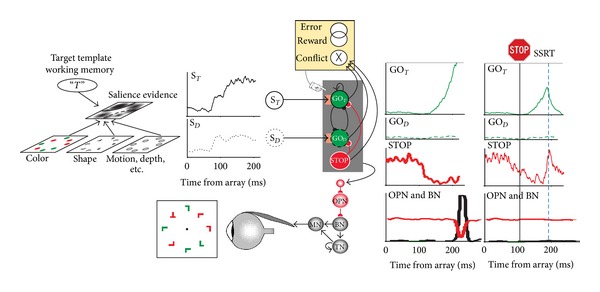
Neural networks for the guidance and control of visually guided saccades. Consider visual search for a red “*T*” among randomly oriented red and green “*L*”s. The color and shape of the objects are specified in feature maps that could also represent motion, depth, and other visual features. These feature maps converge on a map that represents the evidence for salience at each location. This salience map is also informed by a target template in working memory. The timecourse of the salience evidence representation at the target location (*S*_*T*_, solid line) and a distractor location (*S*_*D*_, dotted line) is plotted. According to the gated accumulator model, this evidence is integrated by a network of mutually inhibitory units that will produce a saccade to the target (GO_*T*_, solid line) or to a distractor (GO_*D*_, dotted line). A gate (orange box) prevents integration of noise by requiring the salience evidence to be of sufficient magnitude. A saccade is produced when the activation of a GO unit reaches a threshold (gray horizontal line) at which point inhibition is imposed on omnipause (OPN) neurons (red line) that releases inhibition of burst neurons (BNs) that innervate motor neurons (MNs) to produce a pulse of force to rotate the eye rapidly. The eye velocity signal from the BNs is integrated by a network of tonic neurons (TNs) that also innervate the MN to establish a step of force necessary to maintain eccentric fixation of the target. The activation of the GO units is also influenced by gaze-holding STOP units that release inhibition on the GO units while saccade preparation transpires. If a stop signal of some kind occurs, then the STOP units potently interrupt the GO unit activation from reaching the threshold; this interruption occurs within the theoretical interval known as stop signal reaction time (SSRT) (rightmost columns). An executive control network (yellow) comprised of neurons sensitive to errors, reward, and the conflict arising from coactivation of mutually incompatible response processes signals the consequences and conditions of an action. This executive control network may influence the level of the gate that systematically changes the beginning of the accumulation process to emphasize either speed or accuracy in task performance.

The most direct evidence for a neural instantiation of stopping has been obtained in single-unit recordings from the FEF and SC of macaque monkeys (Hanes et al. 1998; Paré & Hanes, 2003) [59]. The logic of the countermanding paradigm establishes two criteria; a neuron must meet to play a direct and sufficient role in controlling the initiation of a movement. First, the neuron must discharge differently when a saccade is initiated versus when a saccade is withheld because of a stop signal. Second, this difference must occur before the stop signal reaction time, because that is when the act of control is accomplished. Recent researches investigating human brain function during manual stop signal tasks have focused on a circuit involving the right inferior frontal gyrus, preSMA, and the subthalamic nucleus (e.g., [68, 69]). However, numerous other brain regions contribute to inhibiting partially planned movements (e.g., [70]). Firm conclusions in this area of the literature are premature, though, because the functional measures do not have sufficient time resolution. In contrast, single-unit recordings can resolve the timing of modulation at a level necessary to attribute function with more certainty. In FEF, neurons with visual responses but no saccade-related modulation did not satisfy these criteria; they simply responded to the presentation of the stimulus. However, neurons with saccade-related and fixation-related modulation in FEF and SC did satisfy the criteria (Figure 1). After the target appeared, movement-related activity in both structures began to grow toward the trigger threshold. If the stop signal occurred but the activity happened to reach threshold, a noncanceled error was produced. However, successfully canceled trials occurred when the movement-related activity was inhibited, so that it did not reach the threshold activation level. The source of this inhibition appears to be a signal such as that conveyed by fixation neurons in FEF and SC. The pronounced modulation of fixation-related and movement-related activity when saccades were canceled occurred just before SSRT elapsed. The quality of modulation of the movement and fixation neurons is entirely consistent with the fact that movement and fixation neurons in FEF and SC provide direct input to the brainstem structures that produce eye movements (Segraves, 1992) [37].

These results obtained with stop signal task have been replicated in a double-step saccade task with visual search [71] for which performance is the outcome of a race with two GO processes and a STOP process (Camalier et al. 2007). A subsequent analysis demonstrated a quantitative difference between movement and visuomovement neurons (Ray et al. 2009). Movement neurons exhibited a progressive accumulation of discharge rate following target presentation that triggered a saccade when it reached a threshold; if saccades were canceled, this accumulating activity was interrupted at levels progressively closer to the threshold at progressively longer stop signal delays. In contrast, visuomovement neurons exhibited a maintained elevated discharge rate until a brief enhancement announced saccade initiation; if saccades were canceled, the enhancement did not occur. The functional distinction between movement and visuomovement neurons is consistent with recent evidence for biophysical differences as evidenced by spike width ([72]; see also [73, 74]). 

The pattern of results obtained in SC and FEF with the countermanding task is consistent with our best understanding of the functional properties and connectivity of the different neuron types. Thus, the countermanding paradigm is diagnostic of neurons producing signals sufficient to control saccade initiation and thus be said to contribute directly to saccade preparation. Now, neurons in other cortical areas such as SEF and LIP have been described as saccade related (e.g., Schlag & Schlag-Rey 1987; Schall 1991; [75]). This hypothesis has been tested in both areas with the countermanding paradigm, and the results are unambiguous. Vanishingly few neurons modulate before SSRT in SEF (Stuphorn et al. 2010) or LIP [76]. This result indicates terms like “preparation” or even “intention” may not apply usefully to neural activity in SEF or LIP.

### 3.2. Interactive Race Model of Countermanding

The control of saccade initiation is accomplished by interactions between gaze-holding and gaze-shifting neurons. The current data demonstrate this for movement and fixation neurons in the FEF and SC, but it is likely that corresponding neurons in the basal ganglia, thalamus, cerebellum, and brainstem will be modulated in a manner sufficient to be said to control saccade initiation. Does this mean that gaze-shifting and gaze-holding neurons instantiate the GO and STOP processes of the race model explaining countermanding performance? The specification of this linking proposition is not trivial [77]. One facet of this complexity concerns the central assumption of the race model, namely, that the finish times of the GO and STOP processes are independent (Logan & Cowan, 1984). If the neural circuit that instantiates the GO and STOP processes consists of interacting neurons, how can the circuit produce behavior that appears to be the result of independent processes? This paradox has been resolved through a simple network model consisting of one GO unit and one STOP unit (Boucher, et al. 2007; see also [78]). Each unit was a noisy accumulator with RT specified by the time when the GO unit reached a threshold. The network fit the performance data and replicated the form of the activation of movement and fixation neurons if and only if the STOP unit inhibited the GO unit in a delayed and potent fashion (Figure 1). This interactive race has been instantiated in a network of biophysically realistic spiking neurons [79]. 

This fruitful coordination of a task producing a particular pattern of performance, a formal mathematical model, and neurophysiological observations establishes the plausibility of identifying the abstract, formal GO, and STOP processes with the activity of specific neurons. This result validates the utility of SSRT as a measure of impulse control in developmental and clinical studies. However, the mechanistic basis of the potency of the STOP unit inhibition that affords the appearance of an independent race between the GO and STOP processes is not entirely clear. The current evidence emphasizes the contribution of fixation neurons in FEF and SC, but other recent work has demonstrated that neurons in rostral SC contribute to production of microsaccades [80]. Hence, perhaps “stopping” a saccade to a peripheral target is accomplished by producing more microsaccades around the fixation spot. This plausible hypothesis is contradicted by a recent finding of less, not more extraocular muscle activation when saccades are canceled [81]. Furthermore, it seems beyond dispute that some active gaze-holding mechanism exists. Another plausible source for a general gaze-holding signal is the SNpr (e.g., [82]); however, this pathway seems more complex than a simple inhibitory gate (e.g., [83]). Yet another source of inhibition of the neurons instantiating the GO process is local inhibition within FEF, SC, thalamus, and basal ganglia, but how could such intrinsic inhibition be coordinated? 

### 3.3. Executive Control

Executive control will refer to the processes that adapt RT according to the consequences of actions. Recent research on the executive control of saccades has been reviewed [84]. After mastering the countermanding task, adjustments of performance continue ([47]; Nelson et al. 2010). For example, RT varies adaptively with incidental or deliberate variation of the proportion of stop signal trials; RT is delayed as more stop signal trials are encountered (see also [85]).

An extensive body of research with humans has identified areas in medial frontal cortex with executive control (e.g., [86]). Consistent with this framework, in monkeys performing the saccade countermanding task, a variety of patterns of neural activity are observed in SEF and dorsal ACC (Ito et al., 2003; Stuphorn et al. 2000). In both SEF and ACC we found distinct populations of neurons that were active after errors or in association with reinforcement, and in SEF but not in ACC, we also found a population of neuron that was active after successful withholding of a partially prepared movement. These three forms of activation could not be explained by sensory or motor factors. While interpreting signals in ACC in terms of monitoring performance is not novel, this interpretation about SEF was a new perspective. However, this framework has been supported by new evidence from functional brain imaging studies (Curtis, et al. 2005; Nachev, et al. 2005) and effects of lesions restricted to SEF (e.g., Parton et al., 2007; Sumner et al. 2007).

The neurons in SEF and ACC discharging after errors may contribute to the intracranial source of an event-related potential recorded over medial frontal cortex known as the error-related negativity (ERN) (reviewed by [87]) that was the first physiological signature of a supervisory control system. A bridge between the monkey single-unit result and the human ERN has been constructed through a series of studies showing first that local field potentials in ACC and SEF exhibit polarization corresponding precisely to the ERN [88, 89], second that macaque monkeys exhibit an ERN recorded from the cranial surface that is consistent with current sources in medial frontal cortex [81, 90], and third that humans performing the saccade countermanding task exhibit the same form of ERN with a comparable distribution of current sources in medial frontal areas (Reinhart et al. 2011). 

The neurons in SEF and ACC that responded to reinforcement events were more diverse (see also Amiez et al., 2006; Shidara & Richmond 2002). Some responded to a secondary tone reinforcer as well as to the primary juice reinforcer. Others responded only to the primary juice reward both when it was earned and when it was delivered unexpectedly. Still other ACC neurons responded only to noncontingent, unexpected juice reward; some of these also showed an apparent visual response. This pattern of activity resembles the signals produced by brainstem dopamine neurons (e.g., Schultz, 2007). Furthermore, some of the error-related neurons as well as the LFP signaled when earned reward was withheld. The existence of these signals in medial frontal cortex is consistent with models of executive function based on dopaminergic learning signals transmitted to ACC (e.g., [91]).

A third population of neurons in SEF was distinguished from the error and reinforcement neurons (see also [92]). These neurons exhibited elevated discharge rate specifically during stop signal trials in which the saccade was correctly canceled, but the modulation occurred after SSRT, so it cannot be responsible for inhibiting the movement. A comparable signal was also observed in LFP recorded in SEF [89]. An interpretation of the signal produced by these neurons is inspired by the hypothesis that the medial frontal cortex monitors response conflict that arises when mutually incompatible processes are activated simultaneously but cannot both run to completion (e.g., [93, 94]). This hypothesis has been offered as an exclusive alternative to the hypothesis that the medial frontal lobe only detects errors. The existence of distinct populations of neurons signaling error, reinforcement, and putative response conflict indicates that each hypothesis has merit. Of interest, no neurons or LFP have been found in ACC that could signal conflict (Ito et al., 2003) [47, 92]. Based on these results, some have proposed that macaque monkeys do not have the neural substrates necessary to generate performance monitoring ERPs similar to those observed in humans ([95, 96]; but see [97]). However, the presence of all the relevant signals in both single units and LFP as well as a homologue of the ERN calls into question the merits of proposal.

As soon as performance monitoring signals were discovered, their relationship to performance adjustments was explored [87]. This has been tested through intracortical microstimulation of SEF of monkeys performing the saccade countermanding task (Stuphorn & Schall, 2006). Electrical stimulation was delivered simultaneously with the presentation of the stop signal, at a current level well below the threshold for eliciting a saccade. The influence of this stimulation on performance was measured by comparing the fraction of non-canceled trials with and without stimulation. The evidence was quite clear that microstimulation of nearly all sites in SEF improved performance by reducing the fraction of non-canceled saccades resulting in a delayed inhibition function. This was a general effect, occurring for both contraversive and ipsiversive saccades. To determine how the electrical stimulation enhanced monkeys' ability to inhibit saccades, stimulation was delivered on some trials with no stop signal. Stimulation in this context caused an increase in saccade latency; this delaying of the GO process allowed more time for the STOP process to finish first thereby improving performance. 

A recent analysis of the original data from FEF and SC showed how this slowing is accomplished [61]. Stochastic accumulator models account for adaptation of RT to minimize errors and maximize rewards most commonly through changes in the threshold of accumulation that triggers a response (Nakahara et al., 2006; Simen et al., 2006; Forstmann et al., 2008) [98]. However, the systematic delay in response time after stop-signal trials was accomplished not through a change of threshold, baseline, or accumulation rate, but instead through a change in the time when presaccadic movement activity first began to accumulate. This result highlights the subtlety entailed in mapping computational models onto neural processes.

## 4. Guidance of Saccades by Vision and Knowledge

Research on the neural mechanisms of saccade target selection in the context of visual search paradigms used in human studies (e.g., Wolfe and Horowitz 2004; [99]) began 20 years ago (Schall & Hanes 1993) and is now a focus for many research groups. This topic has been reviewed before ([77]; Schiller and Tehovnik 2005; [100]; Fecteau and Munoz 2006; [101–106]), so we will only frame the major issues and highlight more recent findings. 

Research on visual search and saccade target selection can be organized through the concept that search is guided through a salience map (also known as priority map), a spatially organized representation in which bottom-up and top-down influences converge (e.g., [107, 108]) (Figure 1). Salience refers to how distinct one element of the image is from surrounding elements. This distinctness can occur because the element has visual features that are very different from the surrounding (a ripe, red berry in green leaves). The distinctness can also occur because the element is more important than others (the face of a friend among strangers). The distinctness derived from visual features and importance confers upon that part of the image greater likelihood of receiving enhanced visual processing and a gaze shift. In the models of visual search referred to above, one major input to the salience map is the maps of the features (color, shape, motion, depth) of elements of the image. Another major input is top-down modulation based on goals and expectations. The representation of likely targets that is implicit in and dependent on the feature maps becomes explicit in the salience map. Peaks of activation in the salience map that develop as a result of competitive interactions represent locations that have been selected for further processing and thus covert orienting of attention.

Saccade target selection coincides with the allocation of visual attention that has been the focus of considerable research (e.g., [109, 110]). Attentional allocation and saccade production interact variously. Some investigators have explained the connection between saccade production and attention allocation by proposing that the allocation of attention amounts to a subthreshold command to shift gaze. This view is known as the oculomotor readiness hypothesis (Klein and Pontefract 1994) or the premotor theory of attention (Rizzolatti 1983). Although this is an influential hypothesis, many observations are inconsistent with a strict interpretation of it (e.g., [111]), and we will highlight more below. Alternatively, numerous lines of evidence demonstrate that the neural process of selecting a target for orienting is functionally distinct from the neural process of preparing a saccade. 

### 4.1. Neural Processing for Target Selection

A network of structures in the visual pathway contributes to selecting targets for saccades. Neurons in primary visual cortex and extrastriate areas in parietal and temporal lobes represent a variety of more or less elaborated features, surfaces, and objects. But visual processing is not concluded in the parietal and temporal lobes, for extensive convergence of signals from numerous areas occurs in FEF (e.g., [112, 113]) and SC [114]. Although FEF has been identified with an advanced level in the hierarchy of visual areas [115], the latency of visual responses in FEF is comparable to that in, for example, area MT and even proceed the latencies of some neurons in V1 [116]. Moreover, the density of neurons in the supragranular layers that project to area V4 identifies a feedforward connection [117] with terminals on dendritic spines, mainly in supragranular layers of V4 [118]. The influence conveyed by this connection from FEF to visual cortex is a central feature of some network models of visual attention (e.g., [119]).

Extensive research has demonstrated how neurons in cortical areas that represent stimulus features are modulated by target and surrounding nontarget features under various task demands (e.g., [120–128]). Another major input is top-down modulation based on goals and expectations enabled by neural circuits in the frontal lobe (e.g., [129–134]). 

We will suppose that the functional salience map corresponds to a population of neurons that are not intrinsically feature-selective but receive input from feature-selective neurons, so that they signal the location of objects that are the target or are target like in a manner that can be used to guide an action like an eye movement. According to this definition, compelling evidence obtained in multiple laboratories supports the conclusion that the neural representation of the salience map is distributed among multiple cortical areas and subcortical structures including FEF, parietal areas LIP and 7a as well as the superior colliculus, basal ganglia, and associated thalamic nuclei. The heterogeneity of neural function within and diversity of connectivity between these areas makes clear that this salience representation is instantiated by an interconnected circuit built from some but not all of the neurons in these structures. Evidence that the selection process observed in these sensorimotor structures can be identified with a salience representation includes the following observations.

When a search array appears (either by flashing on during fixation or after a previous scanning saccade), activation increases at all locations in the map corresponding to the potential saccade targets. This happens because these neurons are not naturally selective for visual features (but see [135]). Following the initial volley, activation becomes relatively lower at locations that would produce saccades to nontarget objects and is sustained or grows at locations corresponding to more conspicuous or important potential targets (Figure 1). This process has been observed in FEF (e.g., Schall and Hanes, 1993; [121, 136–140]), posterior parietal cortex (e.g., [141–147]), superior colliculus (SC) [148–151], substantia nigra pars reticulata [152], and ocular motor thalamic nuclei [153]. In these studies, monkeys are responding to one among multiple alternatives for the purpose of earning reinforcement, usually with a single saccade. The target selection process has also been observed during natural scanning eye movements (e.g., [125, 126, 154]; Zhou & Desimone 2011). Microstimulation and inactivation have demonstrated causal roles in target selection of FEF (e.g., [155–157]); superior colliculus (e.g., [158, 159]), and LIP (Wardak et al. 2002; [160–162]). 

Manipulations that influence attention allocation in humans influence parallel monkey performance and concomitant modulation of neural activity. For example, when search is less as compared to more efficient because target and distractor stimuli are more difficult to discriminate, then the selection process occupies more time and accounts for a greater proportion of the variability of RT (e.g., [137, 138, 142, 163–165]). The well-known effects of target-distractor similarity on search performance that are expressed in response times and choices by macaque monkeys are paralleled in the magnitude and timing of the visual selection process measured in FEF neurons (e.g., [138]). When the target is more similar to distractors through either feature similarity or recent stimulus history, the level of neural activity in FEF representing the alternative stimuli is less distinct, leading to a higher likelihood of treating a distractor as if it were the target [166, 167]. This parallel suggests that the statement “less efficient allocation of attention” describes a the state of the network in which the activity representing a target and distractors is less capable of being distinguished by either a neurophysiologist or a read-out circuit. Another influence believed to be mediated through the salience map is inhibition of return, the decreased likelihood of directing gaze to a location previously fixated. Neural correlates of this have been described in FEF [46], LIP [168] and SC (Fecteau & Munoz 2005). 

The representation of salience is regarded to guide covert as well as overt orienting independent of effector. The neural selection of the target as a visual location to which to orient attention does not inevitably and immediately lead to re-orienting of the eyes. It occurs if no overt response at all is made [169, 170] or if the saccade is directed away from a color singleton [71, 163]. The selection process occurs as well if target location or property is signaled by through a manual response [157, 171–173]. 

Having identified key nodes in the network representing visual salience, further investigation of the mechanism has been accomplished. All of the results described above were based entirely on modulation of discharge rates of individual neurons. It is clear, though, that saccade target selection is accomplished by pools of neurons [148, 174, 175] and probably entails more than just modulation of spike rate because cooperation and competition between pairs of neurons is modulated during target selection [176]. Indeed, correlation in discharge rates of FEF neurons over longer time scales has been reported even before stimulus presentation [177]. Other researchers have measured local field potentials (LFP) in V4, LIP, and FEF during visual search and attention tasks and described increased coherence in the gamma band between spikes and LFP within and across areas such as V4, LIP, and FEF [125, 143, 178]. Although believed to enhance the representation of attended objects, the functional utility of such signals is not undisputed (e.g., [179]). 

An alternative analysis of LFPs is simply to measure the timecourse of differences in polarization when the target is in or out of the RF. This approach corresponds to the measurement of an ERP on the scalp known as the N2pc, that is, a signature of the locus and time of attention allocation (e.g., [180]). The N2pc has been found in macaque monkeys [164]. Source localization procedures indicate that the N2pc arises from parietal and occipitotemporal sources in humans (e.g., [181]) and macaques [182]. In both efficient and inefficient search conditions, the target is selected significantly earlier in neural spike rate modulation than in LFP polarization [183, 184], and the delay varies with search efficiency. It appears that local processing within FEF mediated by spike rates results in delayed changes of synaptic potentials manifest in the LFP.

### 4.2. Interactions between the Frontal Lobe and Visual Cortex during Target Selection

We have described a target selection process that occurs more or less concurrently in multiple cortical areas and subcortical structures. Recent studies in macaque monkeys have investigated directly interactions between FEF and LIP [143], V4 (Gregoriou et al. 2010; Zhou & Desimone 2011), and inferior temporal (IT) cortex [157, 185] as well as an ERP component recorded over visual cortex that indexes attention [184]. While firm conclusions are premature because results were obtained with different tasks, neural signals, measurement procedures, and areas, some results seem consistent across laboratories. First, when search is inefficient, neural signals of attention allocation in FEF precede those in extrastriate visual areas. For example, a recent study demonstrated that spatial selection of a location in FEF precedes object recognition by IT neurons at that location [185] and the selection in FEF is necessary for detection and identification of the target [157]. Similarly, the target selection observed in spike rate and LFP in FEF precedes the N2pc [184], and the delay between selection in FEF and visual cortex increased with the number of distractor stimuli demonstrating that the delay is not due simply to conduction lags. These results expose a puzzling question—if different times of target selection are measured in different nodes of the network and scales of signal; then, when would we say that attention has been allocated? Given the variation in selection time across neurons even within an area, can we say that the target is selected when the earliest, the latest, or some intermediate population of neurons resolve target location? Such a basic question highlights our profound uncertainty about how signals arise in and are conveyed between the areas representing features, objects, and salience.

This influence of FEF on visual cortex can influence the quality of attentive visual processing [157, 186]. Weak electrical stimulation of FEF influences extrastriate visual cortex activity in a manner similar to what is observed when attention is allocated [187–190]. 

### 4.3. From Salience to Saccade

Explaining how sensory representations lead to accurate movements is a classic problem. One approach to this problem is based on the premise that noisy evidence guiding a response is accumulated over time until a threshold is achieved at which time the response is initiated (e.g., [98, 191]). A recent model inspired by this approach provides an explanation for how signals from neurons that represent target salience can be transformed into a saccade command [55, 56] (Figure 1). The model uses the activity of visually responsive neurons in the frontal eye field representing object salience as evidence for stimulus salience that is accumulated in a network of deterministic accumulators producing saccades to each possible target location to generate accurate and timely saccades during visual search. Response times are specified by the time at which the integrated signal reaches a threshold. The model included leak in the integration process and lateral inhibition between the ensemble of accumulators as well as a form of inhibition that gates the flow of perceptual evidence to the accumulators. Alternative model architectures were excluded because they did not fit the actual distributions of response times nor produce activation profiles corresponding to the form of actual movement neuron activity. At present, this is the only model of visual search that accounts for the range and from of response time distributions [192]. This union of cognitive modeling and neurophysiology indicates how the visual motor transformation can occur and provides a concrete mapping between neuron function and specific cognitive processes. 

The picture that emerges is that the process of visual selection occupies a certain amount of time that can be shorter and less variable if the target is conspicuous, or it can be longer and more variable if the target is less conspicuous. If subjects wish to prevent a saccade to a nontarget stimulus, then the preparation of the saccade can be delayed until the visual selection process has proceeded to a high degree of resolution. Neural activity mediating saccade preparation begins to grow as the selection process is completed and the rate of growth of activity leading to the movement varies apparently randomly such that sometimes gaze shifts sooner and sometimes gaze shifts later. 

### 4.4. Stimulus-Response Mapping

The gated feedforward cascade model assumes that saccade production is guided entirely by the visual salience representation. Thus, errant saccades would be explained by failure to represent evidence correctly. While this has been observed in some testing conditions [166, 167], several other lines of research demonstrate that the salience representation can be correct even if responses are incorrect. For example, in monkeys performing a saccade double step task with visual search, visual neurons in the FEF locate the new location of the oddball in the search array correctly even when monkeys incorrectly shift gaze to the old location [71]. Similarly, when manual response errors occur, the selection process in FEF locates the singleton in the search array correctly [193]. But if the brain located the new location of the oddball correctly, why was an error made? A plausible answer appeals to the hypothesis that the response production stage, even though guided by the perceptual stage, can operate independently of the perceptual stage. Further evidence for this is the fact that these errors can be corrected very rapidly, even before the brain can register that the gaze shift was an error ([194]; see also [154]). 

**Figure 2:**
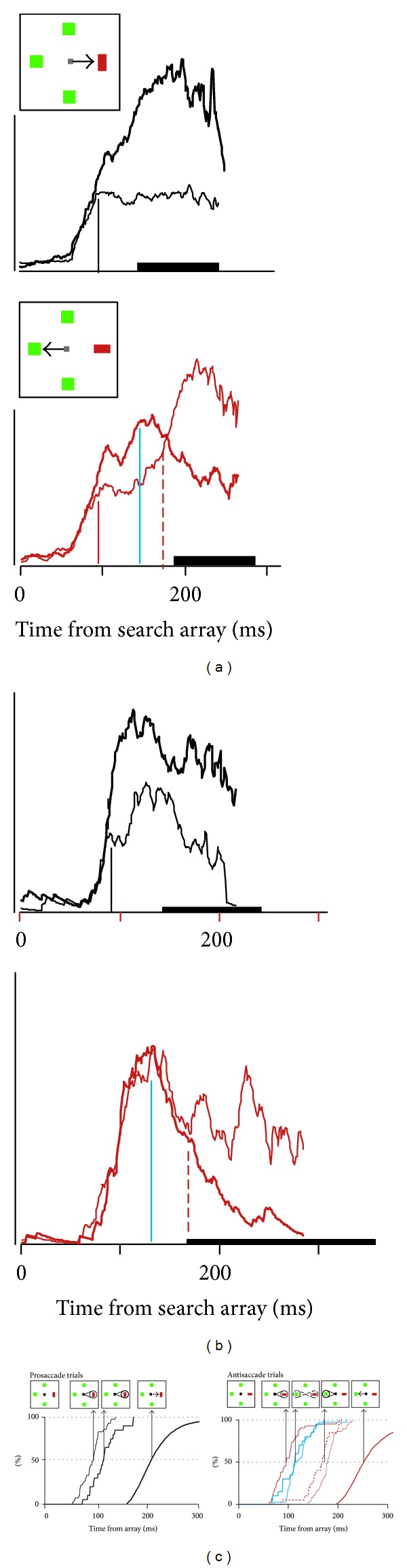
Pattern and timing of neural activity in FEF when mapping between location of visual target and endpoint of saccade is various. (a) Activity of FEF neuron with activity that can be identified with the allocation of attention (Type I). Average spike density function when the singleton fell in the neuron's receptive field (thick line) and when the singleton was located opposite the receptive field (thin line) in prosaccade (top) and antisaccade (bottom) trials. Thick bar on abscissa marks range of RT. Scale bar represents 100 spikes/sec. (b) Activity of FEF neuron with activity that can be identified with selection of the saccade endpoint (Type II). (c) Cumulative distributions of modulation times in prosaccade (left) and antisaccade (right) trials for Type I (thin) and Type II (thicker) neurons with corresponding RT (thickest). The inset arrays indicate hypothesized functional correlates. After presentation of the array, selection of the singleton location occurs first in Type I neurons (indicated by the spotlight on the singleton); this occurs at the same time in prosaccade and antisaccade trials and does not relate to whether or when gaze shifts. In prosaccade but not antisaccade trials, Type II neurons select the singleton at a later time which accounts for some of the variability of RT. A comparison of activation in prosaccade and antisaccade trials reveals the time at which the shape of the singleton is encoded to specify the correct saccade direction; this follows singleton selection and coincides for Type I (thin blue) and Type II (thicker blue) neurons in antisaccade trials. At this moment in antisaccade trials, the representation of the singleton decreases, and the representation of the location opposite the singleton, the endpoint of the antisaccade increases (indicated by the weaker spotlight on the singleton and growing spotlight on the saccade endpoint). At this same time in prosaccade trials, the representation of the saccade endpoint is enhanced by the selection that occurs in the Type II neurons (indicated by the highlighted spotlight on the singleton). Subsequently, in antisaccade trials, the endpoint of the saccade becomes selected more than the location of the singleton by Type I (thin, red, dashed) and Type II (thicker red, dashed) neurons (indicated by the highlighted spotlight on the antisaccade endpoint). The time taken to select the endpoint of the saccade predicts some of the delay and variability of RT. Modified from Sato and Schall [163].

Saccade target selection has also been investigated under conditions that explicitly dissociate visual target location from saccade endpoint. For example, one study trained monkeys to make a prosaccade to a color singleton or an antisaccade to the distractor located opposite the singleton; the shape of the singleton cued the direction of the saccade [163]. As observed in previous studies, the response time for antisaccades was greater than that for prosaccades. A goal of this experiment was to account for this difference in terms of the neural processes that locate the singleton, encode its shape, map the stimulus onto the response, select the endpoint of the saccade, and finally initiate the saccade. Two types of visually responsive neurons could be distinguished in FEF. The first, called Type I, exhibited the typical pattern of initially indiscriminant activity followed by selection of the singleton in the response field through elevated discharge rate regardless of whether the singleton's features cue a prosaccade or an antisaccade. Some of these Type I neurons maintained the representation of singleton location in antisaccade trials until the saccade was produced. However, the majority of the Type I neurons exhibited a remarkable and dramatic modulation of discharge rate before the antisaccade was initiated (Figure 2(a)). After showing higher discharge rates for the singleton as compared to a distractor in the receptive field, the firing rates changed such that higher discharge rates were observed for the endpoint of the antisaccade relative to the singleton location. This modulation could be described as the focus of attention shifting from one location to the other before the saccade. The second type of neuron, called Type II, resembled qualitatively the form of modulation of Type I neurons in prosaccade trials, but in antisaccade trials, these neurons did not select the location of the singleton and instead only selected the endpoint of the saccade (Figure 2(b)). This endpoint selection was distinct from movement neuron activation. The selection times of Type II, but not Type I, neurons accounted from some of the variability of saccade response time on prosaccade and antisaccade trials. 

This experiment revealed a sequence of processes that can be distinguished in the modulation of different populations of neurons in FEF. The timecourse of these processes can be measured and compared across stimulus-response mapping rules (Figure 2(c)). To summarize, Type I neurons selected the singleton earlier than did Type II neurons. In the population of Type I neurons, the time of selection of the singleton in prosaccade and antisaccade trials did not vary with stimulus response mapping or account for the difference in RT. However, the singleton selection time of Type II neurons in prosaccade trials was less synchronized with array presentation and more related to the time of saccade initiation. In antisaccade trials, the time of endpoint selection by Type I neurons was significantly later than that of Type II neurons. This result is as if the endpoint of a saccade must be identified before, attention can shift to the location. The endpoint selection time of Type I neurons in antisaccade trials was too late to explain the increase in RT relative to prosaccade trials. In contrast, the endpoint selection time of Type II neurons in antisaccade trials, like the singleton selection time in prosaccade trials, accounted for some but not all of the delay and variability of RT. The results of this experiment demonstrate that the process of saccade target selection requires a number of representations and transformations beyond simply representing stimulus salience and producing a saccade.

### 4.5. Testing the Premotor Theory of Attention

If shifting visual spatial attention corresponds to preparing a saccade, then it should be impossible to dissociate saccade preparation from the focus of attention even if the endpoint of a saccade is directed opposite the attended stimulus. This was tested by probing the evolution of saccade preparation using electrical stimulation of the FEF [195]. The focus of attention was dissociated momentarily from the endpoint of a saccade by training monkeys to perform visual search for an attention-capturing color singleton and then shift gaze either toward (prosaccade) or opposite (antisaccade) this color singleton according to its orientation [163]. Saccade preparation was probed by measuring the direction of saccades evoked by intracortical microstimulation of the frontal eye field at different times following the search array. Eye movements evoked on prosaccade trials deviated progressively toward the singleton that was the endpoint of the saccade, as expected [196]. Eye movements evoked on antisaccade trials deviated not toward the singleton but only toward the saccade endpoint opposite the singleton. The interpretation of these results is framed by earlier research showing that on antisaccade trials, most visually responsive neurons in frontal eye field initially select the singleton while attention is allocated to distinguish its shape [163]. In contrast, preliminary data indicates that movement neurons are activated but do not produce a directional signal after the saccade endpoint is selected. Evidence consistent with these observations has been obtained in human participants using transcranial magnetic stimulation [197] and in a study probing explicitly the locus of attention [198]. Thus, the brain can covertly orient attention without preparing a saccade to the locus of attention. In other words, target selection and saccade preparation are distinct processes because they can be modified separately (Sternberg 2001). This separate modifiability occurs because different populations of neurons carry out different functions as reviewed above. 

Testing the premotor theory requires specifying the anatomical level at which the mechanism maps onto the brain. If shifting attention is accomplished by the same neurons that are preparing a saccade and if saccade commands are issued by layer 5 pyramidal neurons in FEF and if FEF influences attention by projections to areas V4 and TEO, then numerous layer 5 neurons must be double-labeled by tracer injections in SC and V4/TEO. A recent study found, though, that whereas only pyramidal neurons in layer 5 projected to the superior colliculus, the large majority of neurons in FEF projecting to extrastriate visual cortex are located in the layers 2 and 3, and no neurons projecting to both SC and visual cortex were found [199]. Thus, we can reject the premise that shifting attention is accomplished by the population of neurons that prepare saccades. This conclusion is based on a strict mapping between populations of specific types of neurons and the cognitive processes of attention allocation and saccade preparation. However, a theory formulated too generally to map onto specific neural types loses the relevance of mechanism and force of falsifiability. This result entails that FEF delivers different signals to the visual and ocular motor systems. What, then, is the nature of the influence of FEF on visual processing? If it is not an efferent copy of the saccade command, what else could it be? Anatomical reconstruction of recording sites shows that neurons located in the supragranular layers of FEF are active during the process of attentional target selection [136]. Therefore, the kind of signal that extrastriate cortex receives from FEF corresponds to the target selection process described above. Of course, this is just what is needed to guide the allocation of attention.

## 5. Outlook

This review should demonstrate why researchers in this area feel that steady progress is being made. Looking forward, key questions remain unanswered, though, such as what is the detailed relationship between motor neuron properties, extraocular muscle fiber types, and the forces acting on the eyes? How is the dynamic motor error comparison accomplished? How does preparation of a saccade turn off the OPNs? How can the accumulating activation of multiple, redundant movement neurons be coordinated to produce a saccade at one RT? How are targets for saccades selected? How do multiple, redundant neurons across structures arrive at a single salience representation? Or do multiple salience representations exist in different brain structures, and if so, how are they coordinated? What changes in the representation of salience and preparation of saccades to trade between speed and accuracy? How can the tremendous heterogeneity of neurons be reconciled with the rather limited number of stages and computational processes currently employed to account for performance?

The author is confident that answers can be achieved with effort coordinated across laboratories through complementary tasks and common measurement methods designed to systematically eliminate alternative hypotheses [200] and not contribute to publication bias [201]. The author also believes that answering these and related questions about saccades should not diminish our sense of marvel at the nimble and flexible movements of these shiny globes of gristle.
